# The Technology of Using Liquid Glass Mixture Waste for Reducing the Harmful Environmental Impact

**DOI:** 10.3390/ma15031220

**Published:** 2022-02-06

**Authors:** Viktor Alekseevich Kukartsev, Vladislav Viktorovich Kukartsev, Vadim Sergeevich Tynchenko, Vladimir Viktorovich Bukhtoyarov, Valeriya Valerievna Tynchenko, Roman Borisovich Sergienko, Kirill Aleksandrovich Bashmur, Aleksey Vasilyevich Lysyannikov

**Affiliations:** 1 Department of Materials Science and Materials Processing Technology, Polytechnical Institute, Siberian Federal University, 660041 Krasnoyarsk, Russia; vkukarstev@sfu-kras.ru; 2 Department of Informatics, Institute of Space and Information Technologies, Siberian Federal University, 660041 Krasnoyarsk, Russia; vlad_saa_2000@mail.ru (V.V.K.); 051301@mail.ru (V.V.T.); 3 Department of Information Economic Systems, Institute of Engineering and Economics, Reshetnev Siberian State University of Science and Technology, 660037 Krasnoyarsk, Russia; 4 Information-Control Systems Department, Institute of Computer Science and Telecommunications, Reshetnev Siberian State University of Science and Technology, 660037 Krasnoyarsk, Russia; 5 Department of Technological Machines and Equipment of Oil and Gas Complex, School of Petroleum and Natural Gas Engineering, Siberian Federal University, 660041 Krasnoyarsk, Russia; vladber@list.ru (V.V.B.); bashmur@bk.ru (K.A.B.); 6 Digital Material Science: New Materials and Technologies, Bauman Moscow State Technical University, 105005 Moscow, Russia; 7 Department of Information Technology Security, Reshetnev Siberian State University of Science and Technology, 660037 Krasnoyarsk, Russia; 8 Department of Computer Science and Computer Engineering, Institute of Computer Science and Telecommunications, Reshetnev Siberian State University of Science and Technology, 660037 Krasnoyarsk, Russia; 9 Machine Learning Department, Gini GmbH, 80339 Munich, Germany; roman@gini.net; 10 Department of Aviation Fuels and Lubricants, School of Petroleum and Natural Gas Engineering, Siberian Federal University, 660041 Krasnoyarsk, Russia; alysyannikov@sfu-kras.ru

**Keywords:** AlpHaset system, cold-hardening mixture, foundry production, reclaimed product, sand grain micro-specimen

## Abstract

The spent liquid glass mixture, which is widely used in foundries as a binder after knocking out of moldings, contains pieces of different sizes and strengths, and there is a strong silicate film on the sand grains themselves. The proposed regeneration plants, which provide for the removal of the silicate film by scrubbing, have low productivity and lead to abrasion of the grains themselves. For this reason, the knocked-out mixture is taken to the dump. As a result of the study of the state of the spent liquid glass mixture in the dump, it was found that, in the spent mixture that had lain for 8–10 years, under prolonged exposure to atmospheric precipitation at plus and minus temperatures, part of the silicate film dissolves and almost all monolithic pieces are destroyed. Further use of hydraulic regeneration allows us to reduce the film thickness and thereby reduce the percentage of liquid glass from 5–5.5% to 0.8–1.2%. This made it possible to select the composition of the molding sand for an automatic line, using the AlpHaset-process, which consists of 22–29% of liquid glass mixture from a dump, 65–72% of liquid glass, 5.5% of liquid glass, and a hardener in the amount of 0.55%.

## 1. Introduction

Among all types of inventory production, foundry shows the greatest material use coefficient as reaching 70–90%. Current technology is capable of producing molds with a weight ranging from several grams to 100, and more, tons [1].

At present, foundry production is represented by independent foundry plants, machine engineering enterprise divisions, and metallurgical complexes [2]. Their structures depend on the range of produced castings and scope of production. This determines the selection of the molding technology, such as expendable green sand molds, cold-hardening mixture molds (CHMs), liquid glass mixture molds (LGMs), shell molds, etc. About 67% (in 2006, 67.5% of all castings were made in sandy clay molds, with only 10.2% in molds from cold-hardening mixtures) [3] of all castings are made in green sand molds, while CHMs are used for the production of not more than 12% of the total amount.

Each technology is characterized by its consumption of molding and core mixture, the possibility of their reuse, and the amount of waste mixture taken to the dump.

Ecology remains one of the main problems of the foundry industry, due to the negative impact on human health and the environment, since cast alloy smelting, mold and core production, casting mold pouring and knockout, and casting cleaning emit a significant amount of dust, harmful gases, and solid industrial waste (during work, an emery machine is used, as a result of which dust, gases, and solid waste appear) [4,5]. Modern engineering requires continuous improvement of the technology for producing castings, increasing productivity by shortening the casting manufacturing cycle, improving the surface cleanliness, and significantly reducing unhealthy working operations [6]. The increasing complexity and accuracy, as well as reducing thickness of cast parts, along with the requirements of minimizing labor costs and providing effective environmental protection, significantly affect the development of casting technology. The increasing demands on the quality of castings in the modern world, efficiency of their manufacture, and environmental aspects have led to continuously increasing requirements for the properties of molding and core mixtures, especially in recent years [6].

In order to obtain a casting free of defects, the molding and core mixtures, from which the molds and cores are made, must satisfy a variety of specific properties, since 40–60% of defects in castings are due to the unsatisfactory quality [7] of the molding materials and mixtures. The quality problems of castings have been exacerbated, due to the transition from mass production to small-scale production [8], an increase in the variety and frequency of changes in the casting nomenclature, and the increased environmental requirements for the technological processes used that affect the use of the generated waste.

Environmental problems arise primarily from the casting sand, slag, and dust coming from filtering devices entering landfills. Dust from filtering devices is classified as hazardous waste. From a technical point of view, most foundry waste can be recycled and reused [9].

The disposal of solid foundry waste remains a serious problem [10] for the foundry industry, of which 90% is spent molding and core mixtures belonging to the fourth hazard category. These materials make up the bulk of foundry waste [11].

The most promising areas of development for the foundry industry to reduce environmental risk are the development and assimilation of environmentally safe and waste-free technological processes and equipment, as well as the use of waste mixtures’ regeneration at the places of their formation with a return (up to 95%) into production [12].

In modern conditions, the rational use of raw materials and material resources is of primary importance. Therefore, great attention is paid to the widespread use of industrial waste and related products, instead of primary raw materials [13].

The applied mold and core manufacture technology makes a direct impact on the production costs and labor productivity and, consequently, on the profit and profitability; therefore, it is one of the factors shaping the development strategies of the enterprise as a whole [14]. For this reason, in industrialized countries, cold-hardening mixtures with synthetic resins are attracting more and more attention from the foundry industry [2]. With the high strength of the mixture with small resin consumption comes the possibility of a wide range of mixture hardening speed adjustments, the absence of a drying stage or need for drying equipment, and easy knockout of mixtures from the internal cavities of castings and castings from the molds.

However, the problem that remains to date is the negative impact of the synthetic resin thermal decomposition products on the environment and human safety. From an environmental point of view, foundry and metallurgy comprise of the second-most hazardous industry, after the fuel and energy industries [15]. The worsening environmental condition increases the sickness rate. For this reason, environmental protection is an important factor of foundry industry modernization [16].

Enterprises bear great material costs for both the burial and transportation of molding and core mixture wastes to storage areas [17,18]. The cost induced by the transportation of 1 ton of used mixture to a dump for burial is 6–7 times higher than the fresh molding sand price. This waste contains sand and rock particles, as well as mechanical impurities [16]. Therefore, waste heaps change the structure, physicochemical properties, and mechanical composition of the soil, producing a detrimental effect on the environment of the waste burial areas. The key elements of the waste management strategy are the prevention or minimization of the production of waste, in order to recycle it later; it is suggested that reclamation or destruction of wastes should be applied only when waste reduction is impossible [19]. Therefore, the introduction and implementation of industrial waste production minimization methods are the priority in the waste management strategy.

Recycling 1% of waste reduces the mineral resources production costs by 2%. The exhaustion of mineral resources and need for processing more remote and expensive raw materials are forcing industries to increase the use of man-made mineral formations, which also contributes to the improvement of the environmental situation in the region. This is a solution for two urgent problems: the problem of resources and environment protection [20].

In Russia, the production of 1 ton of iron castings, using resin binders (cold-hardening mixtures), entails the release of the following harmful substances: 10–30 kg of dust; 200–300 kg of carbon monoxide; 1–2 kg of nitric oxide and sulfur; and 0.5–1.5 g of phenol, formaldehyde, cyanides, etc. Moreover, about 3 m^3^ of polluted wastewater flows into the water basin, and up to 6 tons of waste mixtures are disposed into waste dumps [20]. A similar situation can be observed abroad. In India, in the state of Maharashtra alone, the foundry shops of Kolhapur are dumping 700–1000 tons of used molding and core sand per day [21]. Thus, the manufacturing of 1000 tons of castings, with resin binder technology, entails the dumping of up to 6000 tons of the used mixture per year.

Research is aimed to determine the possibility of making a technology for using the waste liquid glass mixture, which will reduce the need for raw materials obtained from quarries, as well as increase production efficiency.

Nowadays, no positive result has been presented on the development of technology that allows for the regeneration of the spent liquid glass mixture (without changing its composition), granting its industrial use. In this work, a technology is presented that allows the regeneration of such a mixture, as well as its use on an automatic molding line operating, using the AlpHaset process, which is the novelty of the research. This will admit businesses to make castings using their own waste, eliminate the heavy manual labor involved in punching out molds, and further increase production efficiency by reducing the cost of purchasing raw materials.

## 2. Review of Today’s Molding Techniques

### 2.1. AlpHaset Mold Manufacturing Technology

Today, it is almost impossible to stop using resin-based, cold-hardening mixtures in the foundry industry [22]. Therefore, one method of reducing the hazardous environmental impact is to create and implement some environmentally friendly binders and cold-hardening mix compositions for casting molds and cores with the same basic physical, mechanical, and technological properties, including preparation technology.

The most widely used mold manufacture technology is the one based on the AlpHaset system, developed by the American company Borden Chemical. It allows for producing high-quality castings from almost any alloys, with low fitting costs and a short manufacturing time, providing high strength of the produced molds and cores [23]. The AlpHaset system was first implemented in the markets of Europe and the USA in 1982 [24]. This technology can be used for the production of ferrous and non-ferrous metal castings of almost any configuration, providing high flexibility in multiproduct goods manufacturing.

The AlpHaset process began to be applied in the market of western European countries in 1982 [25]. In recent years, this technology has spread internationally, especially due to the emergence of new resins and catalysts, which eliminated the unpleasant smell in the molding and embossing areas. This technology has the following advantages [26]:

1. The AlpHaset process allowed for the use of flaskless molding technology, with a flow rate of 2–3 tons of sand per 1 ton of liquid.
2. The percentage of regenerated sand in the sand can be 70–90%.
3. AlpHaset is an efficient and environmentally friendly process of forming and manufacturing cores, which allows for the production of steel, cast iron, and non-ferrous castings, with good surface quality at low labor costs for cleaning and final processing. Thus, the AlpHaset process can be used to replace the casting into shell molds, as well as, in some cases, the exact casting.
4. The easy removal of the rod from the equipment, significant reduction in model wear, and reduction in the pollution and breakage of models make it possible to organize the storage of core boxes and models as close as possible to the place of their use.
5. The AlpHaset binder completely decomposes thermally around the casting when casting bronze, cast iron, and steel; therefore, knocking out forms and rods is easy.
6. There is a minor odor when mixing and filling the equipment, as well as limited toxicity.
7. The water solubility of the binder allows mixers’ cleaning with water.
8. There is weak adhesion to wood and metal (except aluminum) tooling.
9. The storage time of the rods and forms is not limited, and the mixture that already hardens is not hygroscopic.

An essential requirement for the AlpHaset process is low dust content in the molding sand. In other aspects, the process is relatively non-responsive to the fresh sand quality. The effective strength values are achieved with quartz sand, with less than 2% of fine dust particles sized from 0 to 0.125 mm, and a maximum of 5% of the 0.125-mm screen residue [27]. The average sand grain size should range from 0.25 to 0.30 mm. The fresh quartz sand grain shape also has a certain impact on the strength properties. The best AlpHaset molding results are achieved with round and oval sand particle surfaces. Sharp, angular sand grains have a larger specific area, decreasing the mold strength [28].

If these conditions are observed, resin should constitute 1.2–1.3%, and the catalyst should constitute 20–22% of the resin volume. Thus, the arrangement of forced, local, supply, and exhaust ventilation can reduce hazardous emissions in the casting and mold knockout areas, compared to the active resin polymerization process.

### 2.2. Specificity of AlpHaset Technology Use in Russia

Unfortunately, the average grain size of the sand supplied from many Russian sand deposits is 0.20–0.22 mm, the fine dust share exceeds 2%, and the 0.125-mm screen residue exceeds 5%. The analysis, performed by the company Uralchimplast-Kavenagi, in 2012, showed that, for obtaining the appropriate properties, the Russian molding of sand-based AlpHaset mixture contains a 1.8–2 weight fraction of resin and 25–35% catalyst [29]. Therefore, the advantages of the AlpHaset process are reduced, particularly as follows [29]:

1. There is a significant smell, not only in the casting and knockout but also in the molding area;
2. Water rinsing of the mixer is complicated;
3. The half-mold or core removal from the fitting is complicated;
4. Mixture knockout from complex geometry castings is complicated;
5. Due to the increased resin and hardener consumption, the material costs and, therefore, produced casting costs are increasing.

In 2013, the company Uralchimplast-Hüttenes Albertus (UCP-HA) established the manufacture resins required for the AlpHaset process. According to their data, depending on the sand used (provided that the grain particle size is observed), the resin consumption is as follows (in %) [30]:

- Quartz—1.0–1.8;
- Olivine—2.0–2.5;
- Zirconia—0.9–1.3;
- Chromite—1.0–1.5;
- Bauxite—1.0–1.2.

It should be noted [30] that the price of such sands is 10 times higher than that of quartz sand, while the cost of 1 ton of chromite sand can reach USD 800, with that of zirconia sand reaching up to USD 1700.

Moreover, the use of the AlpHaset process does not mean the use of the entire reclaimed product volume, and there is no need for dumping. Use of the regeneration system developed for this process, with some high-quality materials, is a way of reaching a reclaimed product share of 80–85%. This means that 15–20% of the used mixture will be dumped. However, it is necessary to consider spillages and mold defects occurring in the manufacture process, which increases the amount of waste up to 20–30%. This means that the AlpHaset process causes more harm to the environment than the traditional molding production techniques do.

### 2.3. Other Molding Techniques

Another solution to the problem can be the replacement of resin with liquid glass mixtures, based on the CO_2_ process [31]. Such mixtures demonstrate high technological properties and environmental friendliness. This is explained by the fact that casting into liquid gas mixture molds causes the emission of vaporous oxygen and hydrogen only. Nevertheless, liquid glass mixtures have disadvantages, as well. The main negative feature is the high retained strength (strength after mixture heating caused by cast metal effect on the mold and core mixtures). This is the main reason for the hard LGM knockout upon the removal of the core from the casting, as well as the casting itself from the mold. The hard knockout significantly complicates and extends the technological cycle, increasing the net costs and deteriorating the casting quality in a number of cases [31]. Despite this, liquid glass-based molding and core mixtures are still widely used all over the world, since they allow for producing high-quality castings at a relatively low cost.

**Figure 1:**
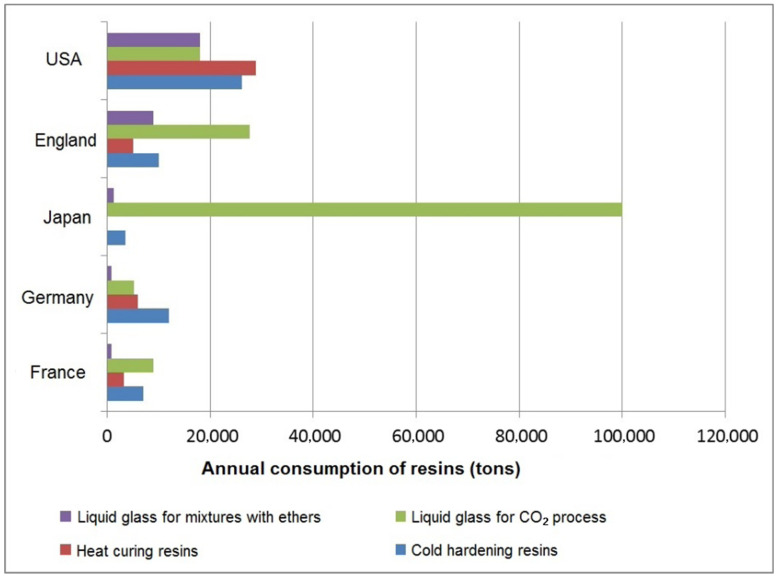
Annual binder consumption in different countries with well-developed foundry production, in tons.

The data shown in Figure 1 show clear evidence of the wide use of form manufacturing using liquid glass [32].

The chemical hardening of liquid glass mixtures, when blowing forms and rods with carbon dioxide (502-process), is based on the property of liquid glass to decompose under the action of even weak acids on it. With the introduction of carbon dioxide into the liquid glass mixture, sodium silicate decomposes, and sodium carbonate is formed with the release of free silica. Silica attaches to itself, presented in the mixture water, and forms a new chemical compound: silica gel. The gel films, located between the grains of sand, bind them into a solid and dry mass. The duration of the purge depends on the dimensions of the form or rod. Thus, for a form with an area of 0.5 m^2^, the duration of purging is 1 min, and for that with an area of 8 m^2^—8 min. Made forms have an average compressive strength of 105 H/m^2^ and tensile strength of 105 H/m^2^.

After knockout from the molding box, the used glass mixture contains pieces of various size and strength, sand (dust), metal impurities, residues of clay, and unburnt coal particles. The sand grains themselves have a strong silicate film [19,33]. This complicates the regeneration of such mixtures and requires their disposal [34].

## 3. Materials and Methods

Research has focused on developing an alternative to using reclaimed water glass mixture from landfills, instead of fresh molding sand.

### 3.1. Used Liquid Glass Mixture Regeneration

The regeneration units (systems), offered by foreign companies, are designed for regeneration of the used CO_2_-treated liquid glass mixture, which contains additives that improve the mold knockout and, accordingly, ensure significant lump destruction on the knockout grid. However, being a part of the regeneration unit, silicate film removal systems do not remove it completely and, moreover, cause the abrasion of the sand grains themselves, disrupting the grain composition.

**Figure 2:**
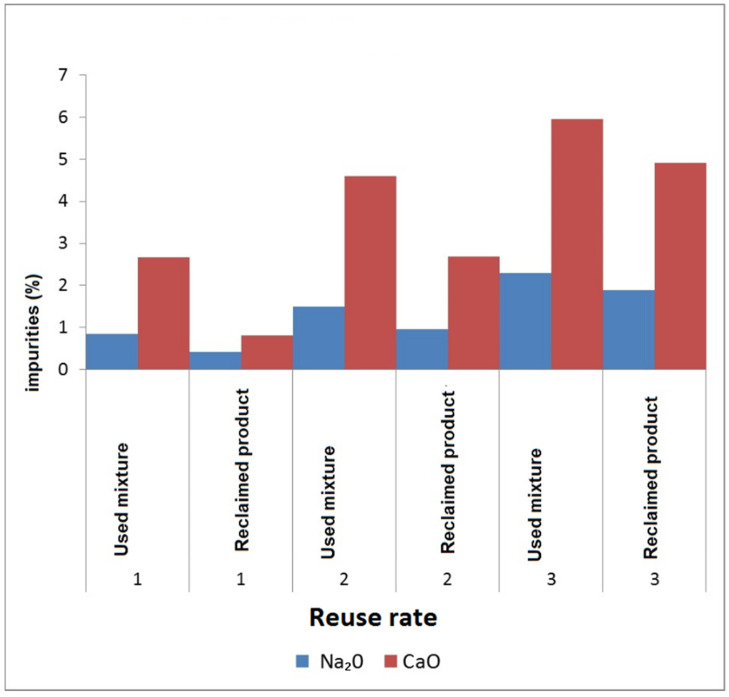
Properties of used liquid glass mixtures.

Russian regeneration units (systems) [35], intended for additive-free traditional liquid glass mixture regeneration, are also incapable of removing the entire silicate film from the sand grains, which was proven by P.A. Borsuk and A.M. Lyass in the 1970s. The data were published after an industrial trial [36] and are presented in Figure 2.

A further reclaimed product reuse rate of four times and more leads to unacceptable saturation of the mixture with the silicate-film-covered sand grains and an increased impurities content (liquid glass should envelop clean sand grains; otherwise, the bonding of sand grains becomes weak, and the molding mixture loses the required strength). The mixture properties deteriorate, increasing the core and mold caused defect rate. Moreover, 15–20% of the used mixture is not subject to reclamation of this kind, as it remains on the knockout grid as large, unbroken lumps and is removed from the process.

### 3.2. Proposed Regeneration Technology for the Used Liquid Glass Mixture

The research involved some specialists from the Siberian Federal University (Krasnoyarsk, Russia). The research included studies of the dumped liquid glass mixture condition, depending on the storage duration, with further wet regeneration.

**Figure 3:**
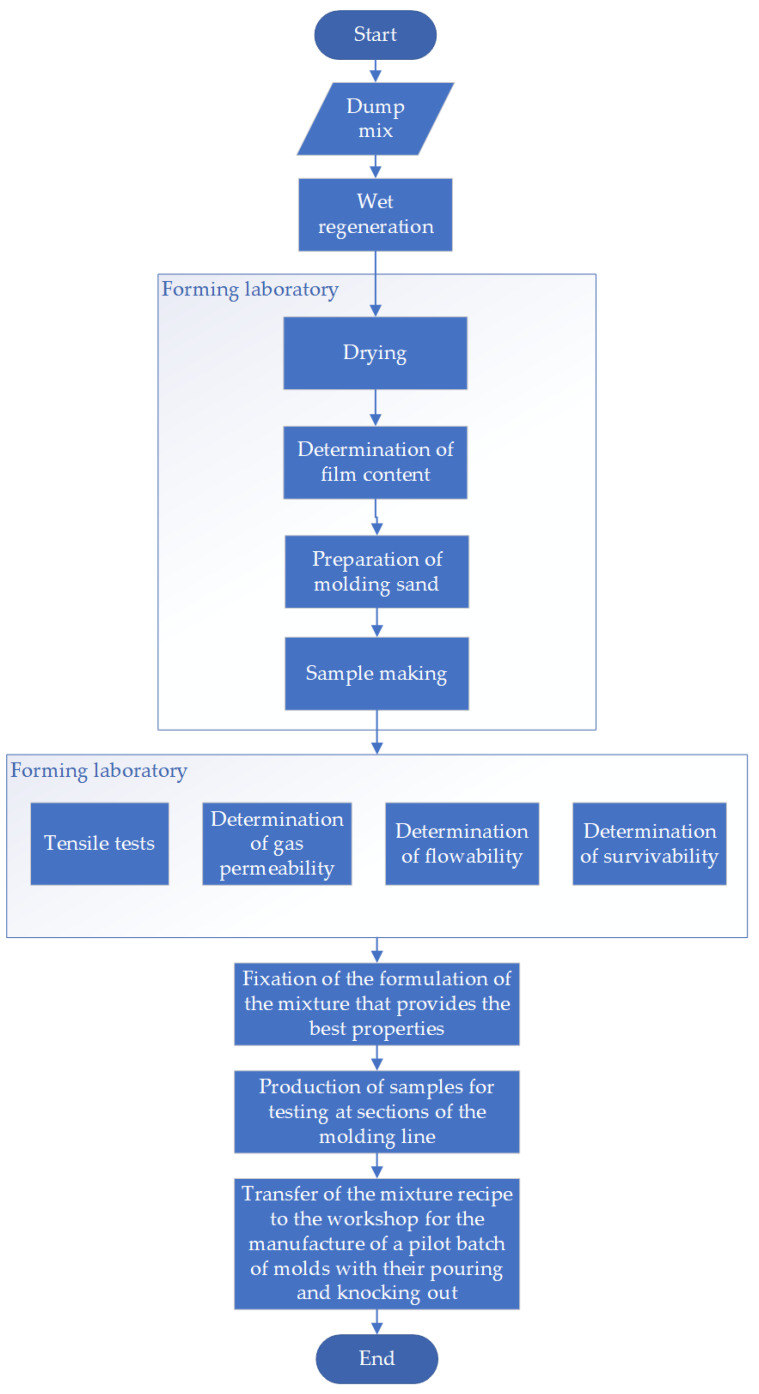
Research algorithm.

The algorithm shown in Figure 3 represents the research process. For the study, samples of the mixture were taken from different parts of the dump; the molding mixture was prepared according to the selected recipe; then, the samples were made and tested for mechanical properties, gas permeability, crumbling, and survivability.

At the foundry of the “Sibinstrem” enterprise (Krasnoyarsk, Russia), for 2 years, work has been carried out aimed at developing a technology that allows for the use of waste liquid glass mixture dumps that have been in open air for 2 to 10 years.

The mixture brought from different dump areas was exposed to wet regeneration; this method did not produce a positive result in using it within a liquid glass mixture composition. Then, the company purchased an automatic molding line from the company IMF, which marked the beginning of the work on the development of forms manufacturing technology [31].

The line required the following tensile strength of the mixture, in kg/cm^2^:

- After 25–30 min: 0.7–0.9;
- After 60 min: 1.3–1.7;
- After 120 min: 2.2–2.7.

The strength of 0.7–0.9 is necessary for proper half-mold removal from the box. If the strength value is lower, the half-mold can fail during the removal; if the strength is higher, the half-mold can get stuck in the box. The strength of 1.3–1.7 is necessary for smooth movement of the manipulator, when transporting and turning the half-molds. If the strength is lower, the manipulator can crush the half-mold; if the strength is higher, the arms of the manipulator may not grip the half-mold, letting it fall or crack, due to a tighter grip. Finally, the strength of 2.2–2.7 is necessary for casting into the completed molds. If the strength is lower, the mold will not be able to bear the pressure of the cast metal [37].

### 3.3. Research Methods and Equipment

The equipment of the molding laboratory was used to determine the possibility of using the spent liquid glass molding and core sand taken to the dump. The equipment is designed to control the prepared composition of the liquid glass mixture used for the manufacture of molds and cores using carbon dioxide blowing. This equipment includes:

- Laboratory runners (mixers) with a bowl capacity of 6 kg;
- Installation for testing the tensile strength of mixture samples, in the form of “eights”;
- Set of laboratory sieves;
- Device for determining humidity;
- Laboratory dryer;
- Device for crumbling determining;
- Laboratory pile driver;
- Laboratory scales.

In addition, in the process of research, the resources of the laboratory were used to determine the modulus and density of liquid glass produced at the foundry. Based on the experience in the preparation and quality control of liquid glass molds and rods, additional devices were used to determine crumbling, survivability, and tensile strength.

Before determining the amount of the waste mixture reclaimed from the dump, it was dried and sieved on a set of laboratory sieves. Sieving was carried out to determine the dust content, on which the required amount of hardener and water glass depended.

The preparation of samples, within the framework of the study, was carried out in four stages:

1. Stirring the reclaimed mixture with water glass and adding the hardener Katasil 1M. Stirring was continued until a homogeneous mass was obtained, which was assessed by color uniformity. The dosage of all components was carried out in measuring containers, with preliminary weighing on a laboratory balance.
2. Pouring the mixture into a multi-slot core box, with internal dimensions that ensure the receipt of samples (eights).
3. Manual sealing of the mixture.
4. Removing samples from the box for subsequent testing at regular intervals, in accordance with the requirements for the mechanical properties of the mixture by the developers of the automatic molding line.

Before each experiment, the ambient temperature and temperature of all components of the future mixture were recorded. Liquid glass was used with a silicate modulus of 2.43 and density of 1.401 g/cm^3^ (based on numerous previous experiments).

## 4. Results and Discussion

### 4.1. Mixture Recipe No. 1

**Figure 4:**
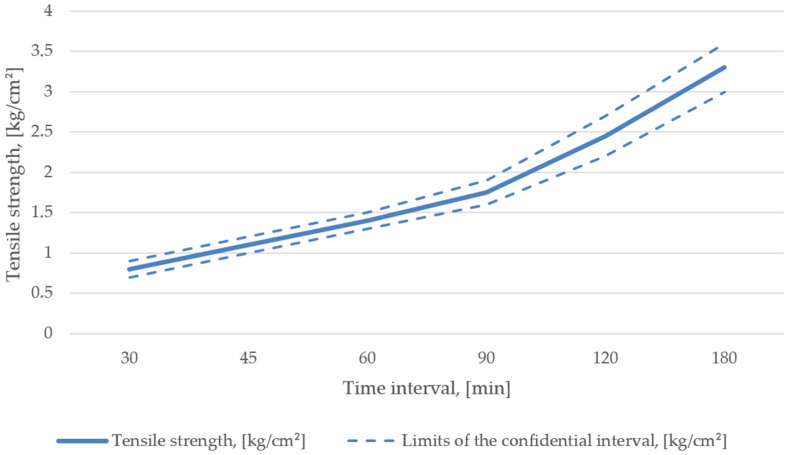
Mechanical properties of mixture recipe No. 1.

During the line set- and start-up, in accordance with the AlpHaset process and 1K20303-type molding sand, it was found that its stability could be reached only with a resin content of 1.8–2.0%, a hardener share of 26–28% of the resin content, and a reclaim share of 76–80% [38,39]. The resultant mechanical properties are shown in Figure 4.

Among the disadvantages of this technology is the strong smell, which caused some workers to retire, regardless of the area where they had been working.

### 4.2. Mixture Recipe No. 2

**Figure 5:**
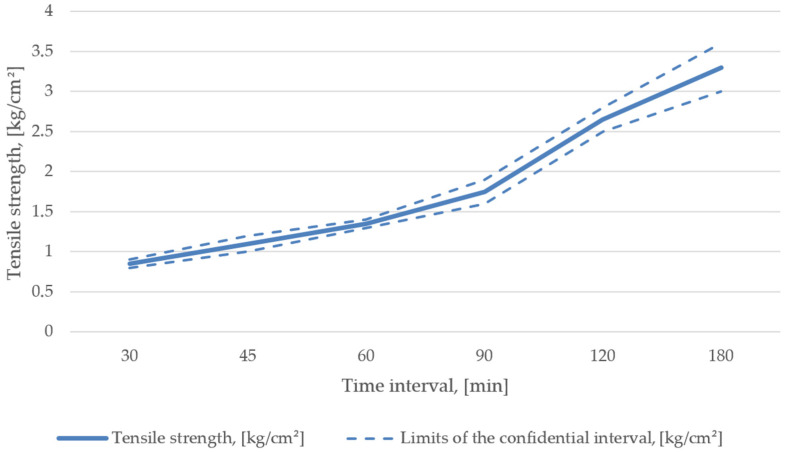
Mechanical properties of the mixture alternative No. 2.

Due to the faults of mixture recipe No. 1, the technology of mold and core production from a cold-hardening mixture, replacing resin with liquid glass, was developed. As a result, the smell was neutralized with the use of 5.5% liquid glass and 0.43% hardener, thus dramatically reducing the raw material expenditure. The mechanical properties are shown in Figure 5.

### 4.3. Mixture Recipe No. 3

In addition, the thickness and amount of liquid glass film on the manufactured microsections were determined by measuring it, as well as through subsequent mathematical calculation. Samples were taken from different parts of the dump, corresponding to the period of its formation.

**Figure 6:**
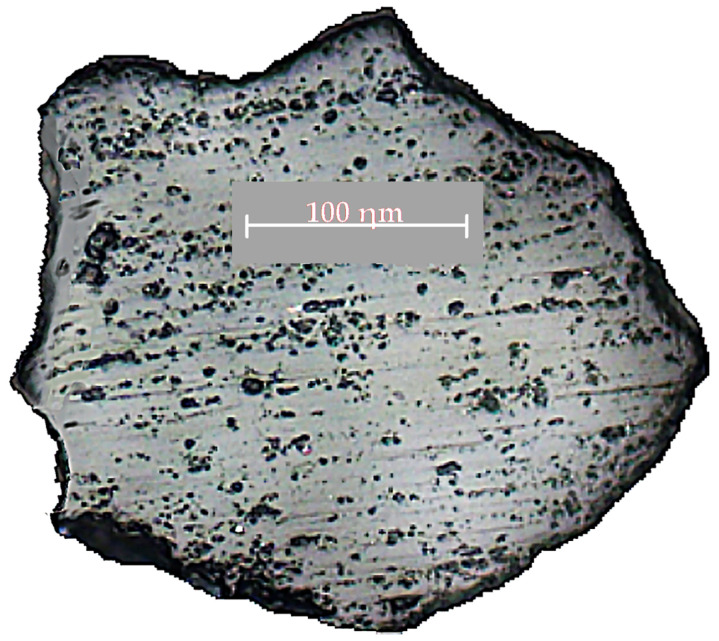
Sand grain micro-specimen from the used liquid glass mixture exposed to long-term outdoor storage.

The study of the prepared micro-specimen, with an Axio Observer A1m inverted metallographic research microscope, showed a film of liquid glass with some bloated areas, as shown in Figure 6. The film thickness was about 0.4–0.6 µm, or 0.8–1.2% of the entire sample surface.

S.S. Zhukovskiy proved [33,40] that with long-term exposure to precipitation at positive temperatures, liquid glass film reacts to water, with part of it dissolving to form different kinds of hydrated metasilicate crystals, including Na_2_SiO_3_*5H_2_O, Na_2_SiO_3_*6H_2_O, Na_2_SiO_3_*8H_2_O, and Na_2_SiO_3_*9H_2_O. In the used mixture, which had been disposed of for 8–10 years, these processes resulted in the collapse of almost all of its monolith pieces.

Nevertheless, as mentioned above, the reclaimed product’s use in the traditional liquid glass mixture did not bring any positive result. Therefore, the next research stage was intended to develop a technology for the regeneration of a used liquid glass mixture, exposed to long-term outdoor storage.

In the laboratory, the mixture was made according to the established recipe; then, samples were made and tested. Upon obtaining satisfactory results, the samples were tested on the production line, with further verification of their properties. After obtaining positive results, the mixture was made already on the line mixer, and further molds were made with their subsequent pouring. In total, approximately 40 different mix formulations were tested.

**Figure 7:**
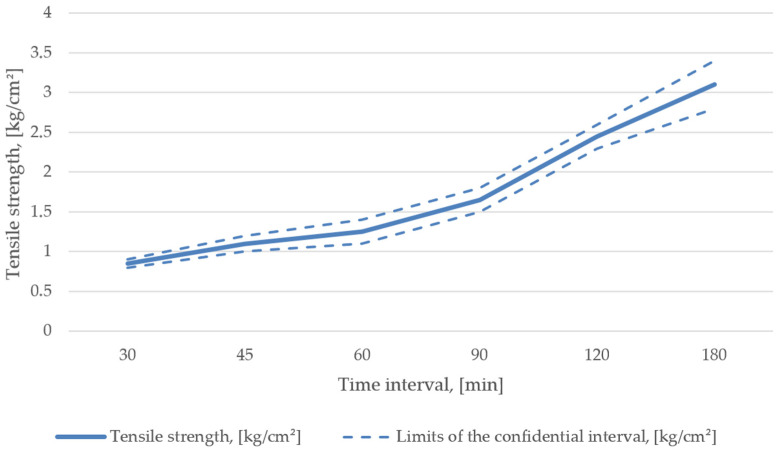
Mechanical properties of mixture recipe No. 3.

Subsequently, the mixture composition consisted of 22–29% of the dumped reclaimed liquid glass mixture, 65–72% of the product reclaimed from the line, 5.5% liquid glass, and 0.55% hardener. The final mechanical properties are shown in Figure 7.

**Figure 8:**
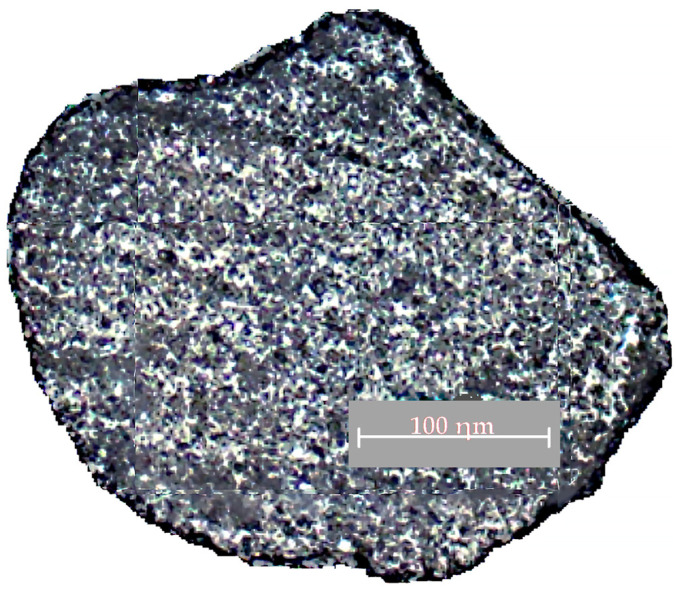
Photograph of a grain micro-specimen from the molding mixture containing the used liquid glass mixture, reclaimed product, liquid glass, and hardener.

Figure 8 presents a photograph of the mixture grain micro-specimen after regeneration.

The film of the liquid glass with the hardener was 0.2–0.25 µm thick, which was about 0.4–0.5% of the whole surface of the sample. This composition was further used for molds manufactured on the automated molding line with the AlpHaset process.

### 4.4. Analysis of the Mechanical Properties of the Mixtures under Study

Investigations into the formulations of the molding sands, using reclaimed product taken from the dump, were carried out in the molding laboratory using the conventional laboratory equipment used in foundries. For this reason, it was necessary to make some changes to the used test method. For example, a multi-slot core box was developed for the production of samples. Then, the strength test was carried out, not in compression but in tension, which was not previously used for such mixtures.

**Figure 9:**
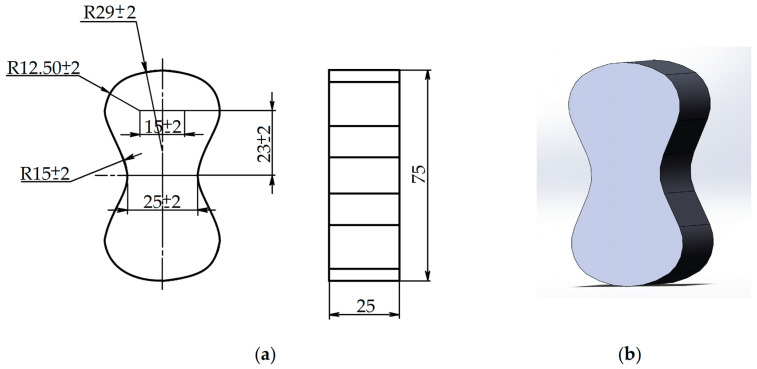
Samples (unit: mm), where: (**a**) designed sample; (**b**) obtained sample.

To prepare the molding mixture, a laboratory casting bowl mixer, with a bowl capacity of 6 kg, was used. First, the refractory filler was mixed with resin, and then a catalyst was added. The mixing continued until a uniform mass was obtained. The components were weighed on laboratory scales and proportioned with scaled containers. The mixture was poured into a multiple core box. Its internal dimensions ensured the production of samples (eights), complying with the requirements of Figure 9a, and with a surface roughness not exceeding Ra = 0.40 μm. The mixture was packed manually. For the subsequent tests, the samples shown in Figure 9b were removed from the box after certain time intervals, according to the requirements imposed on the mechanical properties of the mixture by the automatic molding line developers.

At the request of the line developers, the mixture should have had the following tensile strength (kg/cm^2^): after 25–30 min, 0.7–0.9 MPa; after 60 min, 1.3–1.7 MPa; and after 120 min, 2.2–2.7 MPa. The strength of 0.7–0.9 MPa is necessary for the high-quality extraction of half-molds from the box. If the strength is lower, the half-mold can collapse when removing it. If the strength is higher, the half-mold can get stuck in the box.

The strength of 1.3–1.7 MPa is necessary for the smooth operation of the manipulator during the transportation and tilting of half-molds. If the strength is lower, the manipulator can crush the half-mold. If the strength is higher, the spikes of the manipulator may not hook the half-mold, and it may fall or split when the force to pick it up increases. Finally, the strength of 2.2–2.7 MPa is necessary when pouring assembled forms. If it is lower, the form may not withstand the pressure of the poured metal—we determined this practically, when debugging the technology of making molds and pouring them.

For the first test, the sample was removed after 25–30 min, which is the moment when the half-mold was extracted from the box. The next interval was 60 min, which is the moment when the half-molds were captured by the manipulator for further assembly. The next interval was 120 min for the achievement of the strength required for casting into the complete molds.

As a result of the research, the mixture specifications were developed. The samples made from the mixtures had all the necessary mechanical properties, as listed in Table 1.

Recipe No. 3, proposed by the authors above, was implemented at the foundry (more than 2 years), with a line capacity of 15 molds per hour (mold size 1200 × 800 × 600), which is about 15 tons of mixture per hour (240 tons per day, 5280 tons per month, 63,360 tons per year). Thus, during the time since the beginning of the proposed technology implementation (2 years), 126,720 tons of mixture and 20 thousand tons of castings were produced.

The obtained results make it possible to return to the production of castings waste (generated during the production process) taken to a dump (industrial waste landfill). Thus, the possibility of re-using the spent molding and core sand is provided by the creation of a technology for removing the liquid glass film from the sand grains, without destroying it, in such an amount that the remaining film does not interfere with obtaining the molding and core sand recipe that meets the requirements of the AlpHaset process.

## 5. Conclusions

This study assessed the state of spent liquid glass molding and core sand taken to a dump and left there in open air for 2 to 10 years. It has been established that wet regeneration, provided for a molding sandy clay mixture, partially removes the liquid glass film, and the degree of its removal depends on the shelf life of the mixture in the dump. If the used liquid glass mixture has been stored outdoors for a long period of time, the liquid glass film on the sand grains can be removed by hydraulic regeneration by 85–90%.

Through laboratory studies, carried out in the molding laboratory, the formulation of the molding sand composition with the use of reclaimed product was determined, which provides the necessary properties for the manufacture of molds on an automatic line. The experimental production of molds was carried out on an automatic line, using a recipe determined by research in the molding laboratory. It has been established that the spent molding mixture, which had been in the dump for 8–10 years, can be used to make molds on an automatic molding line using the AlpHaset process technology.

We may conclude that the research has shown the following:

1. If the used liquid glass mixture has been stored outdoors for a long period of time, the liquid glass film on the sand grains can be removed by hydraulic regeneration (by 85–90%).
2. The reclaimed product can be used for manufacturing molds on automated molding lines with the AlpHaset process.
3. The proposed technology is a way of using old, dumped liquid glass mixtures, thus reducing the harmful impact on the environment.
4. The proposed technology is economically efficient, as it reduces the material costs. In this case, the largest cost reduction would occur in the foundries with in-house liquid glass production (as in most foundries using liquid glass mixture molding manufacturing technology).

The use of the spent liquid glass mixture allows us to reduce the use of fresh molding sand, prevent an increase in the area occupied by industrial waste, and improve the state of the ecological environment on them. The proposed use of reclaimed liquid glass mixture allows us to reduce the cost of purchasing and transporting fresh molding sand, thereby increasing production efficiency. Reducing the need for fresh molding sand will reduce its production and increase the resource efficiency of the country’s raw material base.

The introduction of the technology, proposed by the authors, into production used in foundries with automatic molding lines for flaskless molding, which uses the AlpHaset-process technology, will allow:

1. To completely eliminate odor when working on traditional mix recipes: sand, resin, and hardener.
2. To return to the production own waste, stored at the industrial landfill, and thereby reduce the purchase of raw materials.
3. To reduce the need for raw sand, which will reduce the development of quarries and environmental harm.
4. To admit businesses to make castings using their own waste, eliminate the heavy manual labor involved in punching out molds, and further increase production efficiency by reducing the cost of purchasing raw materials.

The future research activities of this project will be focused on the diffusion of the proposed technology to other foundries, which will require additional research to adapt the technology to the specific conditions of existing industries.

## Figures and Tables

**Table 1:** Mechanical properties of molding mixtures made with different recipes with the product reclaimed from the line itself.

Alternative	Composition of the Mixture				Tensile Strength (kg/cm^2^) with the Interval (Min)					
	Refractory Filler	Binder	Hardener	Reclaimed Product (%)	30	45	60	90	120	180
No. 1	Molding sand 1K20303	Alkaset resin NB7	Katalit 3V	76–80	0.7–0.9	1.0–1.2	1.3–1.5	1.6–1.9	2.2–2.7	3.0–3.6
No. 2	Molding sand 1K20303	Liquid glass	Katasil 1M	70–75	0.8–0.9	1.0–1.2	1.3–1.4	1.6–1.9	2.5–2.8	3.0–3.6
No. 3	Reclaimed liquid glass mixture	Liquid glass	Katasil 1M	65–72	0.8–0.9	1.0–1.2	1.1–1.4	1.5–1.8	2.3–2.6	2.8–3.4

## Data Availability

Not applicable.
